# Supplementary material for: Are carbon emissions trading and green financial instruments synergistic? -Comprehensive quantitative research based on content analysis
Source: PLoS One. 2024 Mar 7;19(3):e0298601. doi: 10.1371/journal.pone.0298601 (PMC10919601; doi:10.1371/journal.pone.0298601)
Supplement: S1 Data — (ZIP) [file pone.0298601.s001.zip › Data/地级市Z_F工作报告.zip!/╔╜╢1⁄2/╝├─■╩╨/└┤╫╘╣1⁄2╓┌║┼í╛┬φ┐╦╩2╛▌═°í┐_2010.docx]

各位代表：

　　现在，我代表济宁市人民政府向大会报告工作，请予审议，并请市政协委员和其他列席会议的同志提出意见。

　　一、2009年经济社会发展在应对危机挑战中取得新成绩

　　过去的一年，面对国际金融危机冲击带来的严峻挑战，全市上下在中共济宁市委的坚强领导下，牢牢把握科学发展跨越发展总基调，认真落实国家、省扩内需保增长重大决策部署，积极作为，科学务实，攻坚克难，化危为机，较快实现经济企稳回升，顺利完成"三保三促、一个高于"的任务目标，各项工作取得新成绩。预计全市地区生产总值完成2280亿元、增长12%；财政总收入272.5亿元，其中地方财政收入134.7亿元、增长12.7%；全社会固定资产投资1100.6亿元、增长25%；社会消费品零售总额869.4亿元，增长19.4%；城市居民人均可支配收入16978元，农民人均纯收入6415元，分别增长8.8%和7.5%。一年来，主要抓了以下几个方面的工作。

　　——强化投资拉动，全力突破重大项目。抢抓政策利好机遇，先后争取到国家、省扩内需新增投资项目322个、总投资144.2亿元。全市各类金融机构年末贷款余额1157.5亿元、比年初新增293亿元，增量增幅均创新高。市县两级投融资平台融资规模达180亿元，首期发行10亿元地方债券获得成功。完成技改投资339.6亿元，占工业投资比重达到57.1%。百个重大项目策划招商攻坚行动成效显著，签约投资3000万元以上内资项目904个，实际到位市外国内资金363亿元、增长14.5%；新批办外资项目61个，直接利用外资4.2亿美元、增长7.3%。完成进出口28.9亿美元，其中出口16亿美元、降幅由年初的20.1%收窄到10.7%。全年新开工规模以上项目3491个，其中亿元以上项目205个。总投资过50亿元的台联电新光源新能源系列项目、总投资45亿元的豪德物流项目、总投资20亿元的根本棉业紧密纺项目等一批产业大项目落户济宁；润峰1兆瓦太阳能发电、阳城2×150兆瓦煤矸石发电、太阳纸业20万吨化机浆等45个大项目竣工投产。济徐高速、临菏路济宁段和京沪高铁曲阜站点相继动工，京杭运河航道北延、微山一线船闸和森达美港区建设进展顺利，南水北调东线和治淮东调南下系列工程提速推进。

　　——深度调整结构，培植产业竞争新优势。制定实施四大千亿级产业及六个优势行业调整振兴规划，在全省率先出台《新能源发展规划》，启动化工、食品等新型园区规划建设。设立3亿元中小企业偿贷周转金，组建3家小额贷款公司和一批担保机构。全市规模以上工业企业发展到3763家、比上年增加550家，完成增加值1076.7亿元、增长14.7%，实现利润246亿元、利税400亿元，降幅分别比年初收窄6.2、2.7个百分点。制造业增加值增长17.3%，拉动全市工业增幅10.5个百分点。新能源、新材料、新医药产业成长加速，高新技术产业产值增长19%，占比达到33%、比上年提高2.2个百分点。华勤、如意两大企业集团销售收入过百亿，利税超千万企业增加到260家。山拖、吉客隆等国有企业实现重组合作。基本完成市属困难工业企业改制脱困任务。民营企业发展到2.9万户，增加值达到1032亿元、增长18.5%。新建省级以上重点实验室和企业技术中心13家，新创39个中国驰名商标、著名商标、中国地理标志商标和55个山东省名牌产品，"如意纺"技术荣获国家科技进步一等奖。完成68个重点工业点源治理项目。我市被评为"全省节能工作先进市"。粮食生产克服冻旱灾害实现增产丰收，总产达446.2万吨、增长7%。标准化生产基地发展到348处、327万亩，农产品质量安全示范区增加到151处、74万亩。良种覆盖率达到98%以上。农机总动力增长10%。新增市级以上产业化龙头企业58家。又有101个无公害、绿色、有机农产品获得认证。"十大渔业品牌"创建有新突破，畜禽规模养殖持续扩张，疫病防控和质量检测监管体系更加完善。新增经济林6.7万亩，美国白蛾防治取得阶段性成果。62座小水库除险加固和100座排灌站维修改造项目竣工。济宁曲阜机场吞吐量超过10万人次。微山、汶上跨入全省旅游强县行列。全市共接待中外游客2260万人次，旅游总收入170亿元、增长12.7%。济宁至扬州港内河集装箱航线开通，跃进港区5个千吨级煤码头投入运营，顺利完成34个运河小码头清理任务。济宁商业银行更名为济宁银行。与多家国内知名商业银行达成来我市设立分支机构协议。相继建成运河城财富广场、大润发等新型商贸企业。出台扶持房地产业健康发展政策，商品房销售面积同比增长40.6%。

　　——拉开布局框架，强力推进城镇开发建设。新一轮城市总体规划和土地利用规划修编完成并获省政府批准。适时作出开发建设北湖生态新城重大决策，全面实施U型路、湖底清淤等路网水网管网建设及文教体育设施先导性工程，全年实物工作量达到31.6亿元。高标准完成洸河路、共青团路升级改造，全民健身广场、杨桥三角绿地、百花公园等公共设施建成开放，运河核心景区和金融科技中心、创新大厦等重点工程进展顺利，济北新区建设拉开框架，科技新城一批标志性工程加速崛起，西南片区开发初具规模。济徐高速机场连接线竣工，太白楼路东西延伸及跨河大桥建设进度加快。年初确定的84项城建重点工程有32项基本竣工，完成投资88.6亿元。对城乡主干道、城市出入口等重点区域部位实施高标准"五化"改造，完成中心城区104条背街小巷综合整治任务。城市管理综合执法切实加强。治理车辆超限超载成果不断巩固发展。县域城市和小城镇建设步伐加快、特色凸显，14个新型乡镇进入集约发展新阶段。实施150个新型农村社区和住房建设示范工程。启动环南四湖大生态带规划建设。修复新建人工湿地4.8万亩。治理采煤塌陷地1.6万亩。森林覆盖率和城市绿化覆盖率分别提高到26.5%、41.2%，空气质量优良天数比上年增加17天。

　　——注重统筹协调，力促社会事业全面进步。城乡免费义务教育全面实现。新建改造中小学校舍42万平方米。省级规范化学校增加到104所。高中阶段入学率达到88%。山东理工职业学院、济宁职业技术学院和济宁技术学院新建扩建工程进展顺利。济宁医学院新校区奠基。高等教育毛入学率达到24%。曲阜国际孔子文化节办节水平和综合效应有新提升。开工建设乔羽音乐厅等重点文化设施。一批文艺精品在全省全国获奖。医药卫生体制改革稳步推进。甲型H1N1流感、手足口病防控扎实有效。新型农村合作医疗参合率提高到97.1%，城镇社区卫生服务覆盖率达到95%以上。组织开展"千名科技人员服务企业"活动，与中科院、山东大学等院所共建鲁南工程技术研究院取得积极进展，新建产学研合作基地36家。全年取得重要科技成果180项，申报专利3100件、授权2650件。我市荣获"全国科技进步先进市"称号。加大"百名博士引进工程"和"千名人才引进计划"实施力度，近400名海内外高层次人才来我市创业。以一流的场馆和服务保证了十一运赛事承办的圆满成功。人口和计划生育基层基础工作得到加强。"法治济宁"创建扎实深入。14个单位进入全国精神文明建设先进行列。"双拥"共建和人民防空、国防动员教育、民兵预备役建设在创新中发展提高。第六次人口普查全面展开。工商、物价和食品药品执法监管切实加强。妇女儿童、青少年、红十字会、慈善、老龄和新闻出版、广播影视、档案史志、文博社科等项事业健康发展，防震减灾、水文气象和民族宗教、外事侨务及对台工作取得新成绩。

　　——增进百姓福祉，切实保障改善民生。市财政安排直接关系民生支出9.2亿元、增长21%。为民所办10件实事如期兑现。及时出台实施更加积极就业政策的具体意见，全年新增城镇就业8.7万人，其中下岗职工再就业4.4万人。免费为2.1万名农民工实施技能培训，新增农村劳动力转移就业8.5万人。建起155个见习基地、创业园区，实现高校毕业生就业5万人。养老、失业、医疗、生育、工伤等社会保险体系日益完善，城镇居民医疗保险基本实现全覆盖。城市低保标准由每人每月170元至240元提高到200元至270元，农村低保标准由每人每年900元至1100元提高到1000元至1300元。新型农村养老保险试点工作启动。145家乡镇敬老院全部实现集中供暖。残疾人康复、就业、扶贫各项政策措施落实到位。建成经济适用房24.5万平方米，为4820户城镇困难家庭提供了廉租房，资助4400户农村贫困家庭新建住房1.3万余间。城区新增供热面积300万平方米。为库区移民发放补贴1.2亿元，兑现各类惠农资金8.8亿元。新修农村公路936公里。完成3.4万农户的"一池三改"项目。基层治安防控体系日益健全，打霸除恶"霹雳行动"战果显著。国家安全工作措施持续强化。安全生产形势稳定好转。我市荣获"全国社会治安综合治理优秀市"称号。群众工作服务平台功能逐步增强，各类矛盾纠纷排查调处及时有效，信访责任制和领导干部公开接访制度深入落实，社会应急管理切实加强。对口援建地震灾区马槽乡重点项目如期完成。

　　——加强自身建设，提高行政效能服务质量。扎实开展深入学习实践科学发展观活动，不断深化"三学三创"，各级政府领导班子在解放思想、转变作风、提高公信力执行力方面有新进步。严格落实"三重"工作目标责任，集中会战攻坚行动富有实效。新一轮市县政府机构改革加紧推进，金乡、泗水两县进入"省管县"财政体制改革试点。认真贯彻行政许可法和国务院全面推进依法行政实施纲要，及时向人大报告工作、向政协通报情况，人大代表建议和政协提案办复率均为100%。第7次清理压减市级行政许可项目125项、减幅达到40.6%。市县行政审批中心服务效能稳步提高。土地出让、政府采购等公共资源交易步入规范化、制度化轨道。涉企收费"一费制"改革落实到位。科学民主决策机制更加完善，政务公开针对性、时效性增强，"市长公开电话"倾听社情民意、解决实际问题的质量效率进一步提高。全面落实党风廉政建设责任制，严格遵守各项廉政纪律，深入开展"双评"和纠风专项治理，加大了对行政不作为、乱作为的问责力度。审计监督工作进入全国先进行列。执行厉行节约各项规定，市级行政经费开支比上年压减5%以上。

各位代表！2009年形势复杂多变，挑战空前严峻，困难超乎寻常，我市经济社会发展取得这样的好成绩极为不易，这是全市上下迎难而上、拼搏实干的结果。在此，我代表济宁市人民政府，向全市工人、农民、知识分子和干部，向各级人大代表、政协委员，各民主党派、工商联、各人民团体和各界人士，向中国人民解放军驻济部队指战员、武警官兵和公安干警，向中央、省驻济单位，向支持参与济宁建设发展的港澳台同胞、海外侨胞和国际友人，表示衷心感谢和崇高敬意！

　　在应对危机中直面挑战、砥砺奋进，我们经受了重大考验，获得了深刻启示，也积累了宝贵经验。坚持科学发展跨越发展总基调不动摇，咬定"三保三促、一个高于"任务目标不放松，是我们战胜困难、开拓进取的根本前提和内在动力；锲而不舍推进结构调整，坚定不移加速经济转型，是我们增创优势、赢得主动的现实选择和必由之路；用足用活政策争取倾斜支持，盯住关键环节创新体制机制，是我们化危为机、破解瓶颈的制胜之策和有效之举；聚全市之力苦干实干，以超常规举措会战攻坚，是我们突破重点、培植亮点的重要法宝和可靠保障；困难条件下更加关注民生，尽心尽力为民办实事解忧难，是我们凝心聚力、共克时艰的活力之源和坚实基础。总之，只要我们全市上下团结一致，同心同德，心往一处想，劲往一处使，汗往一处流，就没有克服不了的困难，就没有实现不了的目标！

　　我们也清醒地认识到，全市经济社会发展还存在一些差距和不足。长期积累的结构性矛盾仍较为突出，新兴主导产业成长不快，自主创新能力不强，外向度偏低；资源环境压力增大，发展方式尚未根本转变；现代农业产业体系不够健全，农民持续增收难度加大；县域发展不平衡，一些地方经济总量偏小、可用财力不强；就业形势依然严峻，群众对社会治安、城市管理、环境保护、公共服务等方面还有不满意的地方。政府自身建设存在薄弱环节，少数部门和干部责任意识不强、办事效率不高甚至以权谋私，影响了政府形象和发展环境。对这些问题，我们一定高度重视，采取切实措施加以解决。

　　二、新一年工作的总体要求和任务目标

　　今年是全面完成"十一五"规划的最后一年，也是战胜危机挑战、加速转型发展的关键一年。国内外经济形势总体好于去年，宏观环境变化蕴藏着新的机遇。尽管世界经济复苏进程曲折缓慢、国内经济回升基础尚不牢固、区域发展竞争日趋激烈可能使我们面临一些新的压力挑战，但国家继续实施积极的财政政策和适度宽松的货币政策，调整完善应对国际金融危机一揽子计划，陆续出台强化"三农"基础、激活消费需求、鼓励发展战略性新兴产业等一系列规划方案和政策举措，为我们增强经济内生动力、培植新的增长点带来了重大机遇；我市主导产业调整振兴布局逐步展开，一批大项目好项目陆续进入收获期，城市承载能力和服务功能日趋完善，将为我们乘势快上、转型发展注入新的活力；经过这次危机冲击的重大考验，各级领导班子领导干部思想观念不断更新，发展思路日益拓宽，驾驭市场经济的能力显著提高，为我们应对复杂困难局面、推进科学发展跨越发展提供了强力支撑。我们一定要增强机遇意识、责任意识和忧患意识，始终保持知难而进的勇气、敢为人先的锐气、跨越赶超的志气，顽强拼搏，苦干实干，奋力夺取科学发展跨越发展的新胜利！

　　今年工作的总体要求是：全面贯彻党的十七大和十七届三中、四中全会精神，以邓小平理论和"三个代表"重要思想为指导，深入落实科学发展观，坚持"科学发展跨越发展"总基调，以"经济战略转型"为主线，把经济提质增速发展作为首要目标，突出"转方式调结构、提增速扩总量、优布局谋长远、惠民生保稳定、抓作风强保障"，在继续强化"三农"工作、工业调整振兴、社会事业发展的同时，更加突出培植战略性新兴产业，更加突出发展服务业，更加突出推进新型城镇化，更加突出科技和人才支撑，更加突出生态建设和环境保护，更加突出保障改善民生，更加突出改革创新和对外开放，更加突出县域经济协调发展，力求速度更快、结构更优、质量更高、效益更好、后劲更强，全面完成"十一五"规划目标任务，为打造区域发展新高地奠定坚实基础。主要预期目标安排为：地区生产总值增长12%，地方财政收入增长13%，固定资产投资增长25%，社会消费品零售总额增长16%，外贸进出口和出口、引进市外国内资金和直接利用外资均增长10%，城镇居民人均可支配收入和农民人均纯收入均增长8%，城镇登记失业率控制在4%以内；万元地区生产总值能耗、二氧化硫、化学需氧量排放全面完成"十一五"规划目标，人口自然增长率控制在7‰以内。

　　完成上述目标任务，需要我们把握大局、精心谋划，抓住关键、科学运筹，注重处理好五个方面的关系：一是处理好加速发展与转型升级的关系，继续强化投资拉动、扩充经济总量，更加自觉主动地转变发展方式、提升产业层次，形成在加速发展中提高质量效益、在转型升级中实现跨越发展的良好态势。二是处理好增量优化与存量提升的关系，依托现有基础，大力推进主导产业调整振兴，实施高质高端高效战略，打造新的增长极，靠传统产业焕发生机、新兴产业加速崛起构建现代产业体系。三是处理好重点突破与统筹协调的关系，全力全速攻克重点领域、关键环节，保证"三重"工作任务落到实处，善于把经济发展与城市开发、新农村建设及社会事业、生态文明扭在一起抓，通盘制定落实相互衔接配套的系列政策举措，开创互促并进、协调发展的新局面。四是处理好干好当前与谋划长远的关系，一天也不放松地抓好当前经济运行，加快实现由企稳回升向提速增效的转变；科学编制"十二五"规划，展开新的战略布局，在重大基础设施配套完善、组群结构城市深度融合、主导产业和重点园区集聚发展等方面超前部署、及早实施、务求实效。五是处理好发展经济与改善民生的关系，靠加快经济发展为改善民生提供物质保障，通过落实惠民政策为扩内需保增长增添内在动力，集中更多公共资源向民生领域倾斜，在保证城乡百姓共享发展成果的同时，调动和凝聚起推进改革建设的强大合力！

　　三、坚决打好转方式调结构攻坚战

　　顺应新一轮经济发展新趋势，坚持保增长促发展与转方式调结构的有机统一，着力在提升产业层次和整体素质上下更大功夫，在强化创新驱动和大项目带动上下更大功夫，在培植以低碳经济为特征的新增长点上下更大功夫，走出一条富有特色和竞争力的转型发展之路。

　　（一）加速经济结构优化升级。坚持传统产业改造提升与新兴产业培植壮大一起抓，着力膨胀拳头产品、建设重点项目、扶持骨干企业，加快构建战略性新兴产业为先导、先进制造业和现代服务业为主体的新型产业体系。

　　力促战略性新兴产业加快成长。按照"有限目标、有所作为、突出特色、抢先一步"的原则，加紧编制专门规划，明确年度发展目标，完善配套扶持政策，集中培植新能源、新材料、新医药、新信息及节能环保等产业。新能源领域重点规划建设济宁高新区光电信息产业园，加快台联电系列项目运作落地，支持润峰集团、英克莱扩张产能，鼓励相关企业兴办专业协作项目，争取尽早形成从研发到制造的完整垂直产业链和横向配套产业群，朝着打造"中国光谷"的目标迈进；加快开发车用新型动力电池，鼓励相关企业引进核心技术发展电动汽车。新材料领域依托意可曼、凯伦硅业、泰山玻纤等骨干项目和优势企业，促使功能性高分子材料和新型纤维材料两大拳头产品做大做强；通过现有企业嫁接改造，引进建设离子膜、碳纤维、特种合金等精细化工和高性能金属新材料项目，及早形成特色新材料产业聚集区。新医药领域主攻高端化学合成医药、生物制品、现代中药和医用材料，支持辰欣药业、方健制药等优势企业开展科技攻关、推进合资合作和开发系列新品，逐步向产业链高端攀升。新信息领域主要是深化与各大电信运营商的战略合作，联手实施应用软件开发，培植光通信和电子元器件骨干企业，积极开发高端电子信息产品。节能环保领域主攻节能电子电器、节能建材设备、环保设备和新能源装备的研发生产，支持新风光电子、金曼达电工等企业加速膨胀规模、放大带动效应。

　　提升先进制造业竞争实力。突出抓好煤化工、装备制造、能源工业、食品工业四大千亿级产业培植，落实年度技改投资重点项目和骨干企业扩张目标，力求四大产业对规模以上工业贡献份额提高2个百分点，其中装备制造业争取突破千亿元。实施"千户企业改造提升工程"，抓好纳入国家、省产业调整振兴规划的737个项目，年内技改投资占到工业投资的60%以上。抓好"如意纺"技术产业化、小松行走系统、吉利发动机及零部件、华勤半钢子午胎、太阳高档文化用纸、海螺水泥等重点项目，尽快形成主导产业集群。加紧运作落实民生煤化、中银联想、梁山菱花、中油燃气等一批大项目，打造集聚、低碳、循环、安全的新兴化工园区。大力推进"创百亿工程"，鼓励扶持太阳纸业、华勤集团、山推股份、如意科技、荣信气源等骨干企业壮大规模、晋档升级，进一步扩充50亿级、百亿级企业为主体的强企方阵。建立国有企业外部董事和外派监事会制度，健全经营业绩考核和薪酬分配激励约束机制。深入实施中小企业成长计划，从资金融通、创业指导、技术创新、发展环境等方面加大倾斜扶持。年内全市新增规模以上工业企业400家，新增利税超千万元企业30家。

　　激发服务业发展活力。抓住扩大消费需求有利时机，突出文化、旅游、商贸物流、金融保险和房地产五大领域，促使服务业发展提速、结构提升，年内力争服务业增加值对GDP的贡献份额提高1个百分点以上。文化产业，支持曲阜新区国家级文化产业示范园区完善规划、创新业态、凸显特色、提升水平，引导嘉祥石雕园国家级文化产业示范基地打造品牌、丰富内涵、延伸链条、增创优势。依托运河核心景区培育古运河文化产业带。引进影视、广告、形象设计等创意文化项目，拓展以数字、网络等高新技术为支撑的新兴文化产业领域，借助大型场馆和重大节庆活动发展会展经济、赛事经济和演艺经济。旅游产业，认真落实国家鼓励旅游业加快发展的"意见"，进一步理顺体制、整合资源，集中推进曲阜尼山风景区、汶上-兖州佛教文化旅游带、梁山水浒文化主题公园等重点项目，抓好香格里拉、太阳国际酒店等旅游服务设施配套建设，扶持发展一批新型旅游企业集团。强化对外协作，推介精品线路，拓展客源市场。商贸物流业，加快推进豪德鲁南商贸物流城、瑞中医药物流园等重点项目，完善森达美系列港区配套设施和服务功能，开工建设济宁海天、微山鲁宏、兖州北站等大型物流中心，鼓励优势企业兴办第三方物流。继续引入国内外知名商业品牌和大型商贸集团，兴建更多各具特色的美食、购物、休闲"一条街"。抓好农村供销社改革试点，深入推进"万村千乡"市场工程，支持城市超市、连锁店向农村延伸服务网络。进一步扩大家电、汽车、摩托车下乡覆盖范围和规模，继续实施家电、汽车以旧换新。强化工商、物价执法监管，规范市场秩序。金融保险业，支持市内商业银行深化改革、创新服务，鼓励济宁银行拓展经营领域，促使兴业、恒丰等商业银行入驻我市尽早展开业务。从加强改善管理入手，推动保险业规范健康发展。继续引进创办研发设计、服务外包、电子商务、中介咨询、总部经济等项目，合理布局建设高端服务业聚集区。房地产业，进一步放开市场，引进更多市外大公司和战略投资者参与我市房地产开发，着力培育一批资质等级高、综合实力强的市内建安企业。按照保障性住房优先、普通商品住房放活的原则，持续扩大房地产开发规模，优化商品房供应结构，落实相关扶持政策，支持居民购买自住性、改善性住房。

　　推动县域经济协调发展。坚持促强扶弱带中间，鼓励经济强县放大优势、率先领跑，把功夫下在高起点推进结构调整、提升自主创新能力、强化园区开发和城镇建设、培植优势产业和骨干企业集群上，增强综合实力，走在全省前列。引导中间县自加压力、加速崛起，重点是增加有效投入、引进产业大项目、壮大特色主导产业，夯实基础，膨胀规模，增强后劲，争取在全省排名位次前移。激励经济薄弱县负重奋起、后发快进，着力用好扶持政策、增强内生动力、强化招商引资、主攻工业经济、大办创税项目，力争经济总量、投资规模、可用财力等项指标增幅高于全市全省平均水平，尽快摆脱落后局面。

　　（二）大力发展现代农业。按照统筹城乡发展的要求，创新"三农"工作思路，深度推进农业结构调整，落实各项强农惠农政策，促进农业增效、农民增收、农村经济又好又快发展。

　　提高农业产出效益。稳定粮食播种面积，实施良种、植保系列工程，主攻中低产田改造和高产创建，争取今年粮食再获丰收。以市场为导向集中发展优质、高效、生态种植业，支持精细瓜菜、食用菌、中药材等优势产业产品适度规模经营。以特色品牌为主导规划建设畜牧、渔湖产品标准化养殖基地。扎实推进"林业六大工程"。健全农产品质量检测和安全监管体系，严密落实重大动植物疫病防控措施。年内新认证无公害、绿色、有机农产品80个以上。鼓励各类投资主体兴办设施农业、城郊型农业、观光休闲农业。重点支持30家产业化龙头企业扩张优势产能，发展多种类型的农民专业合作社，引导各类特色产业基地和示范园区与大中城市专业市场、连锁超市建立紧密的营销关系。

　　完善农业基础设施。加紧推进湖西大堤续建、湖内浅槽等大型水利工程，完成华村、龙湾套水库及65座小水库除险加固任务。及早开工南水北调续建配套项目。抓好泗河生态河道建设示范工程。新建改造农村公路400公里、桥梁40座。年内新增"一池三改"3万户，力争秸秆综合利用率达到90%以上。提升农机专业化、社会化服务水平。充实科技特派员队伍，放大农业科技园区示范带动效应。

　　拓宽农民增收渠道。建立健全农民技能培训体系，扶持农村劳动力和返乡农民工就业创业。建立市内重大工程和产业项目吸纳农村劳动力的供求互动机制，促使更多农民就地转移。发挥龙头企业和合作经济组织的聚集作用，引导农民群众进入产业化经营的各个环节。年内新增农村劳动力转移就业7万人，工资性收入占农民总收入的比重提高1-2个百分点。严格监管确保各项惠农补贴及时足额发放。继续做好扶贫和减轻农民负担工作。

　　推进农村制度创新。稳定完善农村基本经营制度，建立健全县乡两级土地承包经营权流转市场。全面推开集体林权制度改革，年底基本完成确权发证。以实施旧村改造、建设新型社区为契机，盘活农村集体资产，兴办二三产业项目，壮大集体经济实力。积极培育村镇银行、小额贷款公司、资金互助社等新型农村金融组织，建设完善农村信用担保体系。

　　（三）加大招商引资项目带动力度。一是增强招商实效。稳步推进招商引资向择商选资转变，集中力量主攻百项重大工业项目、30-50个重大服务业项目和重大基础设施及房地产项目策划招商。组织开展欧美、日韩、港台及长三角、珠三角、环渤海等重点国家和地区系列招商活动，突出加强与世界500强、大型央企及民营巨头合资合作，抓好已落户我市的内外资项目的延伸招商，力争在引进关键部件、核心技术及地区总部、研发机构等方面实现突破。立足保市场、保份额、促回升发展对外贸易，努力增加机电、高新技术产品出口，力促加工贸易转型升级。鼓励有条件的企业建立境外自主营销网络。支持海关、检验检疫等部门完善"大通关"体系。优先进口先进技术装备，增加战略资源进口储备。鼓励大企业开展境外资源合作开发、企业并购及工程承包。二是创新融资机制。深化与各大商业银行的战略合作，组织开展经常性的重点项目对接推介活动，支持各类金融机构持续加大信贷投放。积极培育资本市场，帮助符合条件的企业上市融资。努力扩大地方债券发行规模。鼓励民间资本进入新兴产业、基础设施、公共服务等领域。引导市县融资平台通过运营城市资源盘活存量、吸纳增量、提高效益，实现滚动发展良性循环。在大力培植财源、强化税收征管、壮大财政实力的同时，发挥财政资金吸附引导作用，更加有力有效地支持重大工程项目建设。注重加强政府性债务管理，防范财政风险。三是突破重点项目。落实"项目建设提速年"活动的具体举措，健全项目运作"绿色通道"，在环评、供地、审核、立项等环节简化程序、提高效率。实行重点项目目录管理，亿元以上大项目落实市县领导包保责任制。完善会战攻坚指挥部体制，促使重点项目按时间节点加速推进。围绕国家明确的八个重点投资领域，继续筛选策划大项目，充实项目库储备，形成项目建设的良性循环。

　　（四）完善科技人才支撑体系。在更高层次上实施科教兴市、人才强市战略，突出抓好"四个建设"。一是抓好创新平台建设。强化鲁南煤化工研究院与重点企业集团的联合协作，主动争取承担国家、省重大科技专项。支持鲁南工程技术研究院整合科技资源，启动实施"四个一百"工程，引入更多大院大所，提升自身装备水平和研发能力。加强各类工程技术研究中心、重点实验室、科技工作站等创新平台建设，高标准建设济宁数控技术研发中心，引导重点园区扩充专业孵化器面积，配套建设中试基地。济宁高新区要努力形成高端自主研发与新兴产业成长良性互动格局，及早跻身国家级高新区行列。规范发展技术市场，完善科技成果转化和中介服务公共平台运作机制。办好第10届中国专利高新技术产品博览会。二是抓好企业创新能力建设。鼓励各类企业与高等院校、科研院所建立长期稳定的技术创新联盟，年内新建100家产学研合作创新基地，再建成3个省级以上技术研究中心。大力实施品牌带动和标准化战略，设立"市长质量奖"，再创一批全国全省知名品牌。鼓励大企业领衔联合攻关和引进消化再创新，争取在战略性新兴产业和先进制造业领域集中突破一批共性关键技术。全年取得重要科技成果200项，专利申请量增长10%，国家级高新技术企业达到60家以上，高新技术产业产值增长30%以上、占比提高2个百分点。三是抓好创新型人才队伍建设。继续实施千名人才引进计划，启动"511"海外人才引进计划和国内人才集聚"百千万"工程，千方百计招引高端急需人才。借助市内外高等院校和专业培训机构，有计划地培养专门人才和技能型、实用型人才。完善包括研发经费、工作条件、生活待遇在内的人才激励政策，重奖科技功臣。做好国外智力引进工作，增强"院士济宁行"活动实效。四是抓好创新环境建设。今年市财政科技人才投入1亿元。督促各类企业足额提取科技开发经费。落实好研发投入抵扣所得税政策。积极争取国家、省科技专项资金。着力引进、鼓励发展风险投资和创投基金，吸引外资和民间资本投入科技研发和成果转化项目。深入落实"国家知识产权试点城市"建设各项措施，强化知识产权保护管理。倡树尊重人才、鼓励创新的良好社会氛围，为人尽其才营造更加优越的环境。

　　（五）毫不放松地抓好节能减排。严格执行政策法规，严密落实监管举措，严厉查处环境违法行为，确保如期完成"十一五"约束性目标。一是强化源头管控。分解落实6个重点行业、100家重点企业及省级园区年度控制目标，突出抓好32个重点工业污染点源深度治理项目，重点推广50项节能减排新技术，加快生活垃圾处理厂和污水处理厂提标改造及管网配套工程进度，确保6处截污导流工程建成使用。严格执行新上项目节能评估、环境影响评价和"三同时"制度，杜绝新上高耗能、重污染项目。如期完成落后产能淘汰年度任务目标。二是发展循环经济。重点培育8家循环经济先导示范企业和5家示范园区，拉长延伸循环经济产业链，建成"零排放"绿色企业。对重点行业和企业实行强制性清洁生产审核。在工业、交通、建筑三大领域选择部分企业和单位，先期启动低碳经济试点。大力推广应用LED新光源、电动汽车等先导产品，加速太阳能和建筑节能一体化，年内既有建筑节能改造面积达到60万平方米。三是严格监管执法。定期组织开展"整治违法排污企业保障群众健康"专项行动，加强对企业日常监督检查，强化排污大户、河流断面及城市污水处理厂的在线监测，及时查处偷排偷放行为。有效治理异味污染和汽车废气、建筑扬尘，持续提升城区空气质量。搞好重点耗能单位节能计量监察和能源审计，强化超标准用能惩戒措施。进一步健全节能减排考核体系，严格实行一票否决。四是抓好生态建设。着眼争创"国家生态示范城市"，提速推进环南四湖大生态带工程建设，完成8个人工湿地水质净化工程，新增人工湿地1.5万亩。多措并举治理采煤塌陷地1万亩。年内森林覆盖率提高到27%。

四、加速推进新型城镇化

　　坚持既定城市发展定位、总体思路目标不动摇，全力实施"城镇化追赶战略"，强化中心城区带动、组群结构城市融合和城乡统筹发展，努力走在鲁南城镇带建设发展前列。

　　（一）编制完善城镇建设规划。依据新一轮城市总体规划，高起点编制重大专项规划、城区和重点区域地段控制性详规及年度重点工程建设规划。组织力量编制历史文化名城保护规划。指导各县市编制土地利用总体规划、县域村镇规划。开工建设市规划展示馆。强化规划执法刚性，坚决查处违法违规建设行为。

　　（二）扩张发展中心城区。年内实施86个重点项目，计划总投资318亿元，完成年度投资200亿元以上。在"一城四区"大框架内，按照保续建、保民生、保重点、保产业的要求加快推进工程项目。一是持续增加北湖生态新城项目密度和投资强度。一手抓路网水网、基础设施配套和生态功能组团建设，及早完成15个道路桥梁新建拓展任务，启动供热管网、污水处理厂和河湖水系整治及湿地建设；一手抓文教、商贸、旅游、居住等功能分区重点工程建设，加快市文体中心、市特殊教育学校一期等项目进度，尽快开工市行政服务中心、群众文化艺术中心等项目。二是围绕上水平、出品位、生财富打造东部科技新城。搞好城市开发与新兴服务业发展的有机衔接，加紧建设现代科技馆、佳世客商业中心等重点工程，及早完成洸府河、蓼沟河综合整治及景观带建设。加快济宁高新区与任城开发区、兖州开发区基础设施对接联通，为建设东部高端产业聚集区奠定基础。三是力促济北新区快出形象、形成亮点。加速新327国道路网升级改造，建成商务中心、融鑫大厦等一批标志性建筑，抓好森林公园扩容和功能完善，充分彰显生态宜居风貌。四是港城联动推进运河生态经济区整体开发。先期启动港口、物流、客运等服务业为主体的起步区规划建设，推动与空港片区、嘉祥高新区的融合发展。五是高起点实施老城区改造提升。重点抓好太白楼路升级改造，搞好沿线楼宇及设施有机整合，协调推进运河核心景区建设，切实优化项目布局、集聚商业品牌、带旺人气物流。同步实施运河路、任城路和红星西路的拓展改造，再完成100条背街小巷整治任务。总结推广和谐拆迁成功模式，切实维护被拆迁群众合法权益。

　　（三）促进组群结构城市融合发展。着眼打造城际干线路网，尽快完成太白楼路东西跨河大桥和运河路高架桥建设，启动曲阜高铁片区整体开发，实施兖州火车站扩建工程。加快济徐高速济宁段建设进度，年内确保临菏路济宁段竣工通车，尽早实现高速路网"井"字型贯通。建设京杭运河济宁至东平湖段航道北延工程及疏港道路。抓住太白楼路东西延伸和新济邹路贯通的时机，科学规划布局重点园区和主导产业，引导相关县市区集中实施一批先导性大项目，打造沿路特色产业聚集带。

　　（四）加快县城和镇村开发建设。县城建设坚持旧城改造与新区拓展并重，协调推进基础设施一体化，合理布局建设行政、商贸和住宅聚集区，实现城市容貌改观与人居环境改善的有机统一。突出抓好14个新型乡镇和省级中心镇建设，打造人口集聚、产业集群和商品集散的特色城镇。加紧推行城镇户籍制度改革，完善住房、教育、社保等综合配套政策，促使有条件的农民有序转为城镇居民。全面完成150个新型农村社区市级示范点建设任务，新建农村住房1000万平方米。

　　（五）提升城市经营管理水平。引导城市公用事业转换经营机制，尽早实现绿化、环卫、垃圾污水处理的市场化运营。切实珍惜、合理利用土地资源，完善城乡建设用地增减挂钩制度，加大城镇土地开发收储力度，严格实行经营性用地出让招拍挂。进一步明确各层级管理职责，突出强化街道、社区基础地位，全面推行网格式、精细化执法监管。巩固扩大城乡环境综合整治会战攻坚成果，建立完善长效机制。深入实施畅通工程，为广大市民提供更加安全便捷的出行环境！

　　五、抓好以改善民生为重点的社会建设

　　践行以人为本、民生为先的执政理念，认真兑现落实上级新出台的各项民生政策，集中财力物力实施经济社会发展急需、人民群众期盼的系列民生项目，如期完成今年为民所办10件实事，努力让城乡群众分享更多发展成果！

　　加强就业和社会保障工作。深入落实更加积极的就业政策，围绕重点人群统筹做好就业工作。建立健全公共投资带动就业增长机制，引导劳动密集型企业、中小企业、民营经济及服务业等领域扩大就业容量，通过开发公益性岗位、政府购买岗位援助困难弱势群体就业，继续落实助企稳岗政策稳定就业。扎实推进"国家级创业型试点城市"创建工作。采取专场推介、择优选聘、"三支一扶"、开辟见习基地和创业园区等措施，鼓励大学生到城市社区、农村基层、中小企业就业和自主创业。全年新增城镇就业7万人，高校毕业生就业率达到85%。加强社会保险扩面征缴，抓好城镇非公有制经济从业人员、灵活就业人员和农民工参保工作。兑现企业退休人员基本养老金人均增长120元的新标准，及早实现社会化养老。尽快将破产企业退休人员和困难企业职工纳入城镇职工医保体系。年内城镇居民医保补助标准提高到100元、新农合人均筹资标准提高到120元。扎实推进新型农村社会养老保险工作，争取扩大试点范围。进一步提高城乡低保水平，其中农村低保标准提高到每人每年1200元至1400元。农村五保集中和分散供养标准分别提高到每人每年2700元、1680元。实施贫困残疾儿童康复救助计划，重度残疾人每人每月增加不低于50元的生活补贴。加快市县社会福利服务中心建设。发挥红十字会、慈善等机构在社会救助中的积极作用。编制实施保障性住房建设三年规划，用好住房公积金增值收益支持保障性住房建设。年内建设经济适用住房36万平方米，新增廉租住房7.8万平方米，改造农村危房1.5万户。继续做好援疆工作。确保对口援建北川三年任务两年基本完成。

　　繁荣教育卫生文化体育事业。立足促进教育公平和均衡发展，突出抓好中心城区中小学校规划建设，合理调整布局，优化资源配置，年内开工济宁一中新校区等一批重点项目。校企合作抓好实训基地建设，提高职业技术院校办学质量效益。促进济宁学院完善装备设施、优化专业设置，支持驻济高校突出内涵发展、建设优势学科，争取济宁医学院新校区和山东理工职业学院实现秋季招生，年内普通高校在校生力争突破10万人。完善助学政策，对中等职业学校农村家庭贫困学生和涉农专业在校生免除学费。强化基层医疗卫生服务体系建设，巩固扩大乡镇卫生院和村卫生室改造建设成果，争取上级扶持启动县级医院改造提升工程，继续实施基本和重大公共卫生服务项目。有序推进基本医疗保障制度改革。强化甲型H1N1等重大疫情防控措施。重视支持中医药事业发展。完善计划生育利益导向机制，稳定低生育水平。构建完善市县乡村四级公共文化服务网络，启动孔子博物馆、市图书馆新馆等重点文化设施项目。支持文艺团体放大品牌效应、推进改革发展。全面完成乡镇综合文化站建设任务。深入实施农村电影放映、广播电视"村村通"、农家书屋建设等文化惠民工程。推行文化市场综合执法改革，净化网络文化市场。筹备办好曲阜国际孔子文化节系列活动。积极推进中华文化标志城规划建设。提高重点文物和非物质文化遗产开发保护水平。重视发展哲学社会科学。落实建设体育强市的关键措施，贯彻实施《全民健身条例》，全力备战22届省运会，积极申办23届省运会。

　　切实维护社会和谐稳定。层层落实维护稳定第一责任，突出抓好社会矛盾化解、社会管理创新和公正廉洁执法三项重点任务，深入落实"平安济宁"建设各项措施，增强人民群众安全感。积极构建打防管控一体的社会治安防控体系，依法防范和严厉打击各类违法犯罪活动。坚持完善领导干部下访和公开接访制度，深入排查调处各种矛盾纠纷，健全社会稳定预警和应急管理机制，超前防范、妥善处理各类突发公共事件。继续创新加强群众工作，保证城乡百姓合理诉求有序表达、实际问题有效解决。深入开展"加强安全生产基层基础建设年"活动，层层落实安全生产责任制，深化隐患排查和专项治理，坚决遏制重特大事故发生。开展食品药品安全专项综合整治行动，真正让百姓吃上放心食品、用上安全药物。巩固提高"双拥"共建成果，加强国防教育动员、人防民防和民兵预备役建设。进一步做好国家安全、民族宗教、外事侨务、对台事务、水文气象、防震减灾、妇女儿童和老龄工作。

六、强化政府自身建设和管理创新

　　面对复杂局面、繁重任务和新的机遇挑战，我们要以更高的标准、更严的要求，进一步加强思想作风建设，努力提高决策能力、执行能力、创新能力、管理能力，引导政府工作人员争做"爱读书、肯实干、操守正"的新型干部，更好地担负起推进科学发展跨越发展的历史重任。

　　强化理论武装，开辟思想解放新境界。持续抓好对科学发展观的学习，切实把学习成果转化为谋划发展的正确思路、促进发展的政策措施、领导发展的实际本领。始终与中央和省、市委保持高度一致，确保各项方针政策和重大部署在政府工作中贯彻执行。结合编制"十二五"规划，加强对宏观经济重大战略问题的研究分析，科学谋划转型发展新方略新思路。以学习促进新的思想解放，打破一切陈旧模式和条条框框，善于在变化转折中抢抓机遇，在攻坚克难中开拓创新，在争先进位中谋求突破。

　　切实改进作风，实现服务效能新提高。分解落实"三重"目标责任，坚持深入建设发展前沿亲历亲为，务求把问题解决在一线，把任务落实在一线，把经验推广在一线。完善科学民主决策机制，重大战略、重要事项主动听取专家和群众的意见建议。规范政务公开内容形式，认真落实服务承诺制，优化市县行政审批服务中心业务流程，巩固扩大涉企收费"一费制"改革成果。加大效能监察和行政问责力度，严肃查处不作为、乱作为行为！

　　推进依法行政，力促政府职能新转变。自觉接受人大法律监督，认真执行各项决议决定，支持人民政协履行政治协商、民主监督和参政议政职能，及时办理人大代表建议和政协提案，充分听取各民主党派、工商联和无党派人士的意见，注重发挥工青妇等人民团体的作用。继续办好市长公开电话，主动接受新闻舆论和社会公众监督。在完成机构改革、落实"三定"规定的基础上，突出抓好政府职能转变，强化社会管理、公共服务职能，同步推进事业单位改革。继续深化新型乡镇配套改革。严格依照法律规定的权限和程序行使权力、履行职责，落实行政执法责任制，加强行政执法队伍建设，确保公正、文明、严格执法。

　　坚持从严治政，树立清正廉洁新形象。注重加强道德修养和党性锻炼，严格执行廉洁自律各项规定，提高拒腐防变能力。全面落实党风廉政建设责任制，强化对领导班子领导干部的教育、管理和监督。建立完善内容科学、程序严密、配套完备、有效管用的反腐倡廉制度体系，努力实现权力公开透明运行。切实强化审计监督。支持纪检监察机关依法查办案件，纠正侵害群众利益的不正之风。发扬艰苦奋斗、勤俭节约优良传统，争做清廉型干部，创建节约型机关。

　　各位代表！新的一年蕴含新的机遇，新的征程充满新的希望。任何困难都动摇不了济宁人民奋发图强的坚定意志，任何挑战都阻挡不了孔孟之乡跨越发展的坚实步伐。让我们在中共济宁市委的正确领导下，高举中国特色社会主义伟大旗帜，解放思想，务实创新，团结拼搏，苦干实干，为推进科学发展跨越发展，创造济宁更加美好的明天而努力奋斗！
